# Macrophage migration inhibitory factor activates inflammatory responses of astrocytes through interaction with CD74 receptor

**DOI:** 10.18632/oncotarget.13739

**Published:** 2016-12-01

**Authors:** Yu Su, Yingjie Wang, Yue Zhou, Zhenjie Zhu, Qing Zhang, Xuejie Zhang, Wenjuan Wang, Xiaosong Gu, Aisong Guo, Yongjun Wang

**Affiliations:** ^1^ Key Laboratory of Neuroregeneration, Ministry of Education and Jiangsu Province, Co-innovation Center of Neuroregeneration, Nantong University, Nantong 226001, PR China; ^2^ Department of Rehabilitation Medicine, Affiliated Hospital of Nantong University, Nantong 226001, PR China

**Keywords:** MIF, spinal cord, astrocyte, inflammation, CD74

## Abstract

Astrocytes, the major glial cell population of the central nervous system (CNS), play important physiological roles related to CNS homeostasis. Growing evidence demonstrates that astrocytes trigger innate immune responses under challenge of a variety of proinflammatory cytokines. Macrophage migration inhibitory factor (MIF), a proinflammatory cytokine mainly secreted from monocytes/macrophages, is involved in inflammation-associated pathophysiology. Here, we displayed that expression of MIF significantly increased following spinal cord injury, in colocalization with microglia and astrocytes. MIF elicited inflammatory responses of astrocytes *via* activation of CD74 receptor and extracellular signal-related kinase (ERK) pathway. Transcriptome analysis revealed that inflammation-related factors cholesterol 25-hydroxylase (Ch25h) and phospholipase A2-IIA (Pla2g2a), downstream of MIF/CD74 axis, were potentially implicated in the mediating inflammatory response of astrocytes. Our results provided a new target for interference of CNS inflammation after insults.

## INTRODUCTION

Astrocytes, together with microglia, are the principal CNS sources of innate immune responses by producing inflammatory mediators such as complement components, IL-1β, IL-6, and chemokines including CCL2, CXCL1, CXCL10 and CXCL12 [1–5]. Astrocyte-specific inflammatory signaling contributes to a multitude of CNS pathologies. Suppression of inflammatory signaling in astrocytes results in improved recovery from spinal cord trauma, and lessened inflammation in EAE, a rodent model of the human inflammatory demyelinating disease [6, 7]. As the major glial cell within the CNS, astrocytes constitutively express an array of receptors involved in innate immune reactions, including Toll-like receptors (TLRs), nucleotide-binding oligomerization domains (NOD), double-stranded RNA dependent protein kinase, scavenger receptors, mannose receptor and components of the complement system [8]. Thus, the cells are highly susceptible to the stimuli of foreign infections or endogenous tissue-injury products, and as a consequence, leading to magnified inflammatory outcomes.

Macrophage migration inhibitory factor (MIF), a small secreted protein of 12.5 kD, was first lymphokine shown to prevent the migration of macrophages out of capillary tubes [9]. Not limited to the production of activated lymphocytes, MIF is ubiquitously expressed by a variety of other cells including monocytes/macrophages, fibroblasts, insulin secreting β-cells of the pancreas, pituitary cells and endothelial cells [10]. Many studies have established that monocytes/macrophages are the primary site of MIF production after stimuli with microbial components and several cytokines [11, 12]. Growing evidence indicates that MIF functions as a proinflammatory cytokine, and as a hormone involved in inflammation-associated pathophysiology such as systemic infections, sepsis, autoimmune diseases, cancer, and rheumatoid arthritis [13, 14]. In the traumatic spinal cord, expression level of MIF increases in the microglia accumulating in the lesion epicenter three days after SCI [15]. Deletion of MIF attenuates neuronal death and promotes functional recovery after compression-induced spinal cord injury in mice [16]. However, it remains unclear whether astrocytes participate in the MIF-mediated neuropathology.

MIF-induced signal transduction is initiated by interaction with the extracellular domain of CD74, the cell-surface form of the MHC class-II-associated invariant chain [17]. Studies of intracellular signaling events have shown that MIF induces rapid and sustained phosphorylation and activation of the ERK1-ERK2-MAPK pathway [10, 18]. In addition, the chemokine receptors CXCR2 and CXCR4 can also recognize MIF, which form a receptor complex with CD74, to activate the intracellular signaling that results in regulation of inflammatory cell recruitment and other biological activities [19, 20]. In the present study, we examined the expression of MIF in the injured spinal cord of rat. We further analyzed MIF-induced inflammatory signal pathway in the astrocytes. Our results indicate that MIF functions on neuropathology through activation of inflammatory signaling in the astrocytes.

## RESULTS

### The expression of MIF significantly increased following spinal cord injury

**Figure 1:**
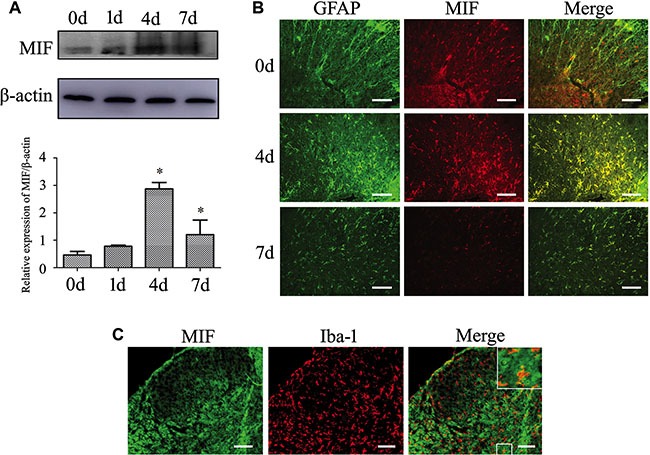
Expression of MIF increased following spinal cord injury (**A**) Western blot analysis of MIF expression following spinal cord contusion at 0d, 1d, 4d and 7d, respectively. (**B**, **C**) Immunostaining showed colocalization of MIF with GFAP- and Iba-1- positive cells. Rectangle indicates region magnified below. Quantities were normalized to endogenous β-actin. Error bars represent the standard deviation (*P* < 0.05). Scale bars, 100 μm.

To understand the function of MIF on neuropathology, we firstly examined its expression changes after spinal cord contusion. Western blots revealed that the expression of MIF remarkably increased at 4d and 7d after injury (Figure 1A). Immunostaining showed that MIF colocalized with IBA-1-positive microglia and GFAP-positive astrocytes, the principal sources of CNS innate immune responses (Figure 1B, 1C). Notably, the expression of MIF in the astrocytes was markedly enhanced with the time extension of spinal cord injury, indicating that the production of MIF seems synchronous with the activation of astrocytes (Figure 1B).

### MIF was able to activate the inflammatory responses of astrocytes

**Figure 2:**
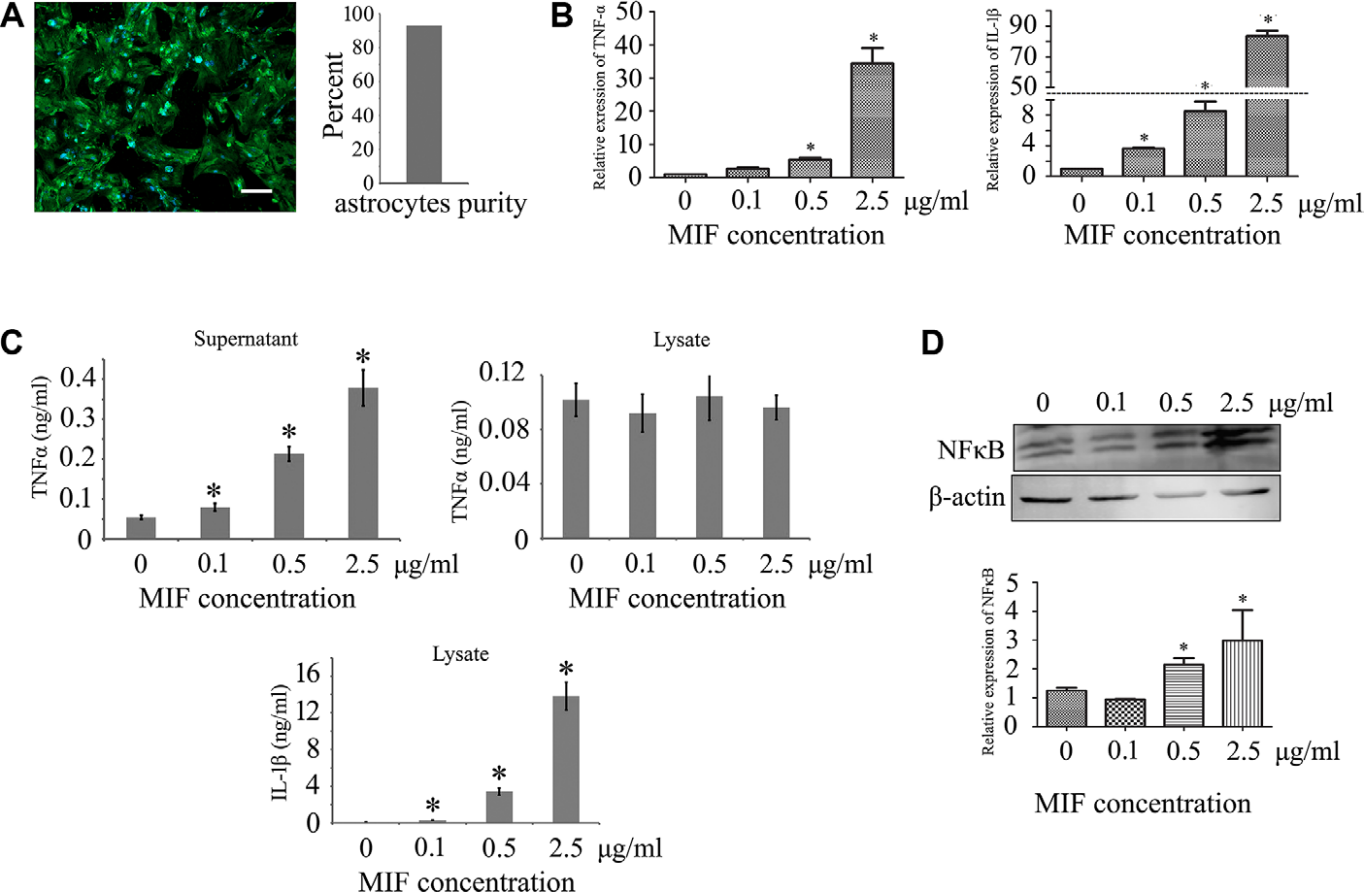
Examination of inflammatory activation in astrocytes following stimuli with gradient recombinant MIF (**A**) Showing purified primary astrocytes stained with GFAP and Hoechst 33342. (**B**) Expression analysis of *TNFα* and *IL-1β* by RT-PCR, following astrocytes treatment with 0–2.5 μg/ml recombinant MIF for 24 h. (**C**) Cell supernatants and lysates were tested by ELISA for the cytokines TNFα and IL-1β. (**D**) Western blot analysis of p65NFκB activation in astrocytes. Quantities were normalized to endogenous β-actin. Error bars represent the standard deviation (*P* < 0.05). Scale bars, 50 μm.

Extensive studies have emphasized the roles of MIF on the monocytes/macrophages, as well as various tumor cells [11, 21, 22]. MIF potently activates intracellular signaling of target cells by both paracrine and autocrine action [12, 23]. To uncover the effects of MIF on the inflammatory responses of astrocytes, we purified primary astrocytes and detected the expression of TNF-α and IL-1β at both transcriptional and translational levels following stimuli with recombinant MIF (Figure 2A–2C). RT-PCR analysis demonstrated that the expression of *TNF-*α and *IL-1*β significantly increased in a concentration-dependent manner after cell treatment with 0–2.5μg/ml recombinant MIF, with IL-1β showing a robust response (Figure 2B). We further tested the production of TNF-α and IL-1β in astrocytes by ELISA. The TNF-α secretion in the cell supernatants increased following stimuli with recombinant MIF, whereas IL-1β accumulated in the cell lysates, rather than in the supernatants (undetectable), suggesting a distinct mechanism of secretion (Figure 2C). The expression level of NFκB, a major transcription factor that regulates genes responsible for both the innate and adaptive immune response, increased remarkably in response to the treatment of different concentration of MIF (Figure 2D), indicating that the effects of MIF on astrocytes were mediated through activation of NFκB signaling.

### MIF interacts with CD74 surface receptor of primary astrocytes

**Figure 3:**
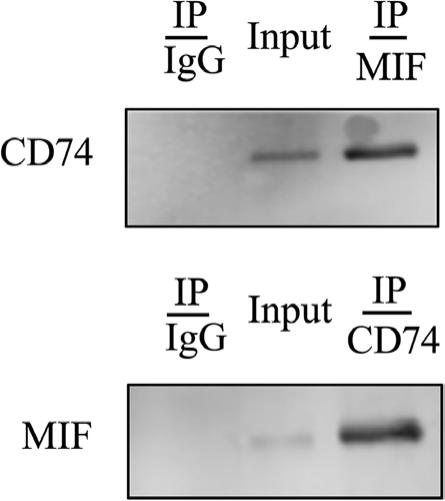
Binding assay of MIF with CD74 receptor in the primary astrocytes Immunoprecipitation using anti-MIF or -CD74 antibody and detection of the components of the MIF- or CD74-associated complexes with anti-CD74 or -MIF antibody.

MIF has been shown to trigger intracellular signaling through binding with CD74 surface receptor, which forms a receptor complex with chemokine receptors, CXCR2 and CXCR4 [17, 19]. To clarify whether such interaction exists in astrocytes, co-immunoprecipitation was applied to assay MIF/CD74 couple. As shown in Figure 3, MIF was present in the CD74-associated complexes when immunoprecipitation using anti-CD74 antibody. So was CD74 when immunoprecipitation using anti-MIF antibody. The results indicate that MIF initiates intracellular signaling through binding with CD74 surface receptor of astrocytes.

### MIF-stimulated ERK activation in astrocytes is dependent on CD74

**Figure 4:**
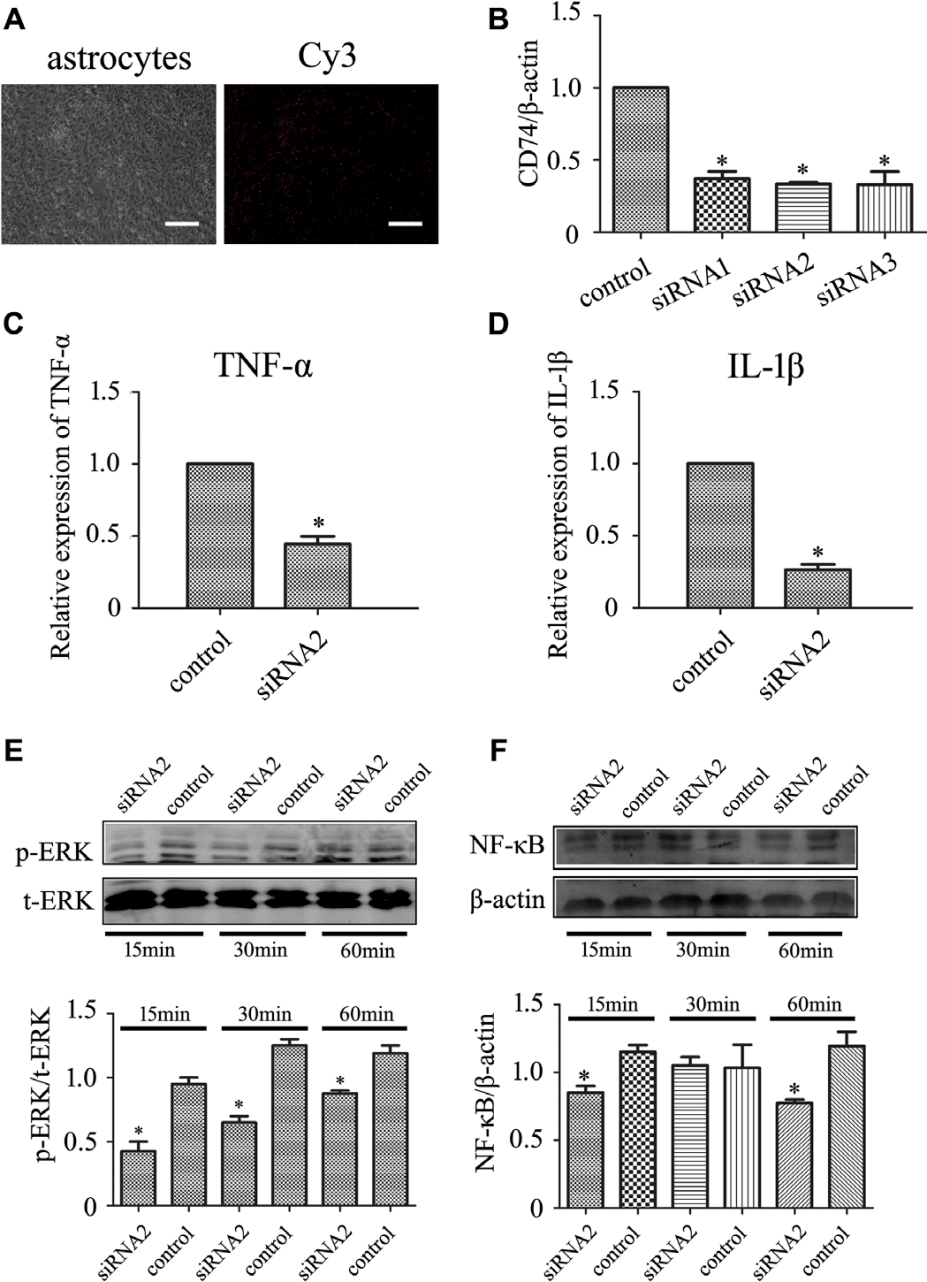
Knockdown of C74 receptor affected MIF-induced inflammatory responses in astrocytes (**A**) Determination of siRNA transfection efficiency by Cy3 control. (**B**) Interference efficiency of three siRNA oligonucleotides for CD74 was measured by RT-PCR, and siRNA2 was used for the knockdown experiments. (**C**, **D**) Expression analysis of *TNFα* and *IL-1β* by RT-PCR, following astrocytes treated with siRNA2 oligonucleotides or scramble for 48 h, and then with 2 μg/ml recombinant MIF for 24 h. (**E**, **F**) Western blot analysis of pERK and p65NFκB following astrocytes treated with siRNA2 oligonucleotides or scramble for 48 h, and then with 2 μg/ml recombinant MIF for 15 min, 30 min and 60 min, respectively. Quantities were normalized to endogenous β-actin. Error bars represent the standard deviation (*P* < 0.05). Scale bars, 10 μm.

MIF interaction with CD74 is necessary for the sustained activation of ERK1/2, which activates I-KappaB Kinase-α (IKKα) in cytoplasm, leading to the phosphorylation and ubiquitination of IκBα, then leading to the translocation of NFκB to the nucleus [24, 25]. To unveil the mechanism of MIF-stimulated inflammatory response in astrocytes, we interfered with the signal pathway through knockdown of C74 receptor. The transfection efficiency measured by Cy3 control experiments was approximately 95% (Figure 4A), and the siRNA oligonucleotides (siRNA2) with the highest interference efficiency of rat CD74 was selected (Figure 4B). Cells were transfected with siRNA2 oligonucleotides for 48 h, and then treated with 2 μg/ml recombinant MIF for 24 h. Interference of CD74 resulted in a significant decrease of *TNF-*α and *IL-1*β expression in comparison with those of scramble (Figure 4C, 4D). Accordingly, activation of p-ERK and the downstream NFκBp65 were attenuated following MIF treatment at 15 min, 30 min and 60 min (Figure 4E, 4F). An asynchronous decrease of p-ERK and NFκB appeared at 30 min, suggesting signal(s) beyond CD74 receptor might be associated with the activation of NFκB. The data indicate that MIF-stimulated ERK activation and inflammation in astrocytes are dependent on CD74 receptor.

### MIF facilitates proliferation of astrocytes

**Figure 5:**
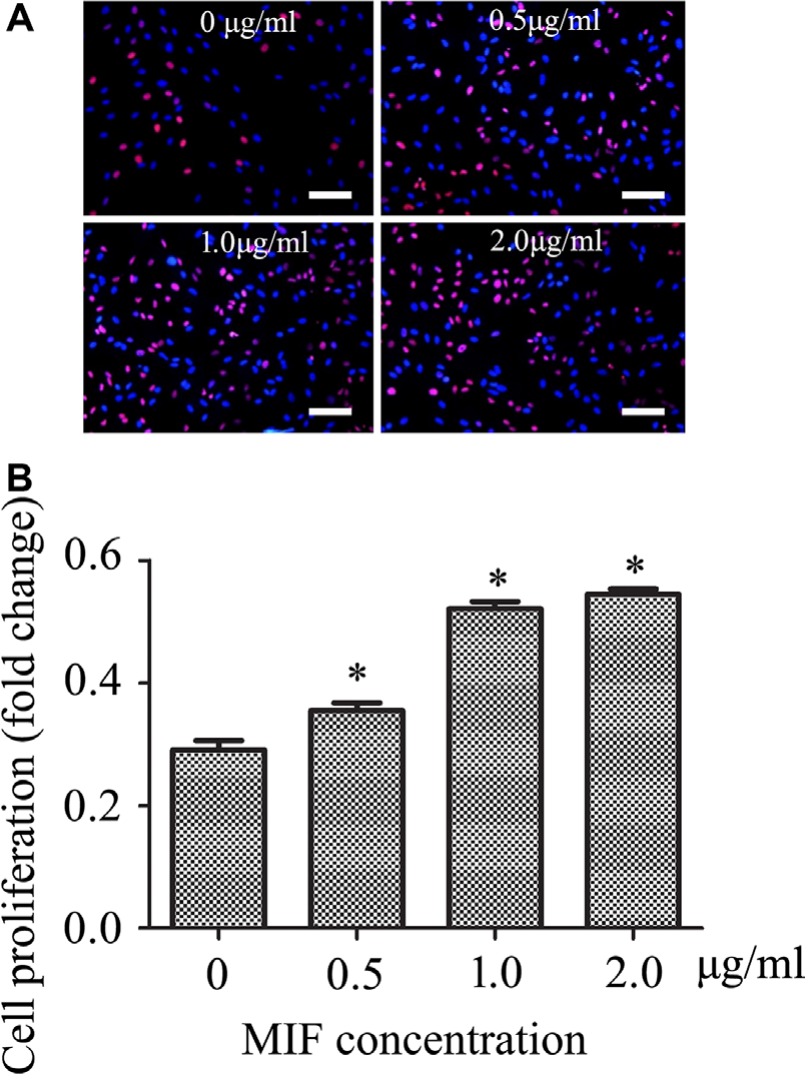
MIF stimulates astrocytes proliferation *in vitro* (**A**) Effects of MIF on proliferation of primary cultured astrocytes. (**B**) Quantification data as shown in (A). Error bars represent the standard deviation. *P < 0.05. Scale bars, 100 μm.

Activation of ERK signaling has been shown to facilitate cell proliferation in carcinogenesis and angiogenesis [26]. To further validate the proliferative effects of MIF on astrocytes, the primary cultured astrocytes were treated with 0, 0.5, 1.0 and 2.0 μg/ml recombinant MIF for 24 h. A remarkable increase in the cell proliferation rate was observed in the presence of MIF (Figure 5A, 5B). These results indicate that MIF facilitates proliferation of astrocytes, in addition to mediating inflammatory response.

### Identification of inflammation-related factors downstream of MIF/CD74 axis in astrocytes

**Figure 6:**
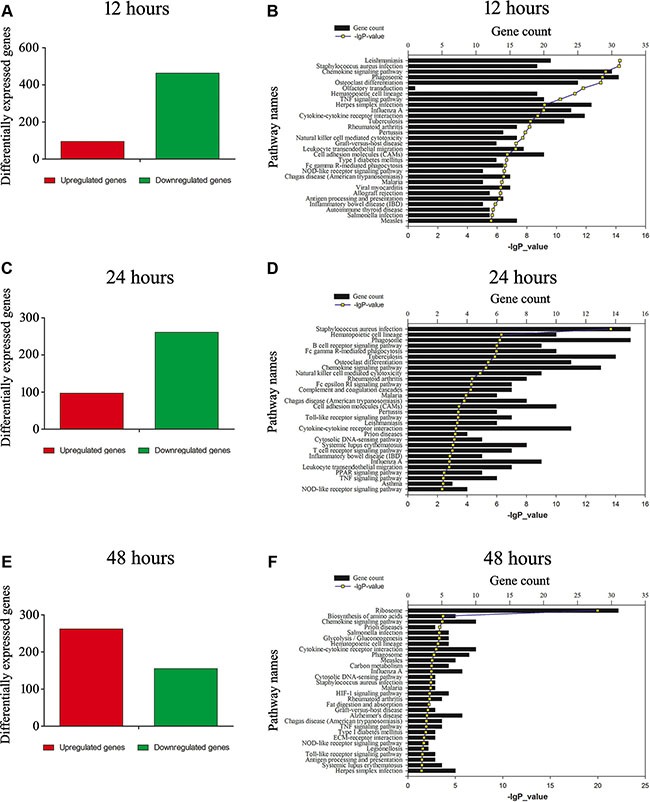
Functional annotations of DEGs in the astrocytes following knockdown of CD74 (**A**), (**C**) and (**E**) Bar graphs of DEGs following knockdown of CD74 for 48 h, and then treated with 2 μg/ml recombinant MIF at 12 h, 24 h and 48 h, respectively. (**B**), (**D**) and (**F**) Most significantly enriched groups for the DEGs relating to pathways.

**Figure 7:**
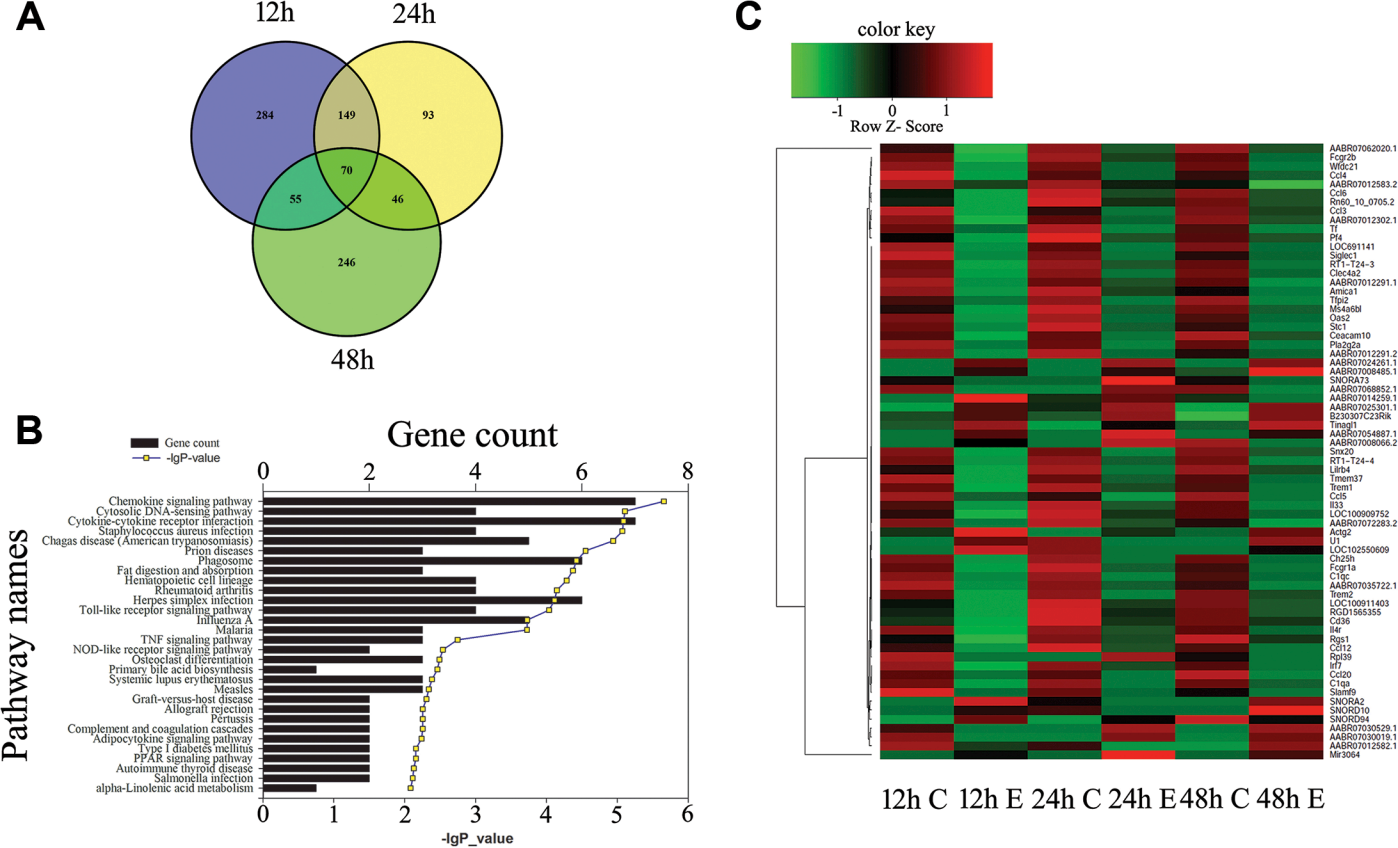
Expression profiling of integrated DEGs in the astrocytes following knockdown of CD74 (**A**) Integration of DEGs at 12 h, 24 h and 48 h. (**B**) Most significantly enriched groups for the integrated DEGs relating to pathways. (**C**) Heatmap and cluster dendrogram of integrated DEGs.

Astrocyte has been regarded as active players in the CNS innate immunity, hinting at the complex regulatory mechanisms for the inflammation and immune reactivity [8]. To identify inflammation-related factors downstream of MIF/CD74 axis, we performed transcriptome analysis on astrocytes treated with 2.0 μg/ml MIF for 12, 24 and 48 h, after knockdown of CD74 siRNA or scramble for 48 h. A total of 558, 358, 417 differentially expressed genes (DEGs, siRNA *versus* scramble) were identified at different time points, with defined criteria of *P* < 0.05 and a greater than twofold or less than twofold changes (Figure 6A, 6C, 6E). KEGG pathway enrichment analysis identified that inflammatory and immune regulation, chemotaxis signaling were included in the significantly enriched functional pathway (Figure 6B, 6D, 6F). We further integrated the DEGs at 3 time points, and characterized 70 functional genes (17 upregulated and 53 downregulated) associated with important biological processes including inflammatory and chemotaxis regulation (Figure 7A, 7B). These genes displayed dynamic alteration following interference of CD74 siRNA, as shown by Heatmap and cluster dendrogram (Figure 7C).

**Figure 8:**
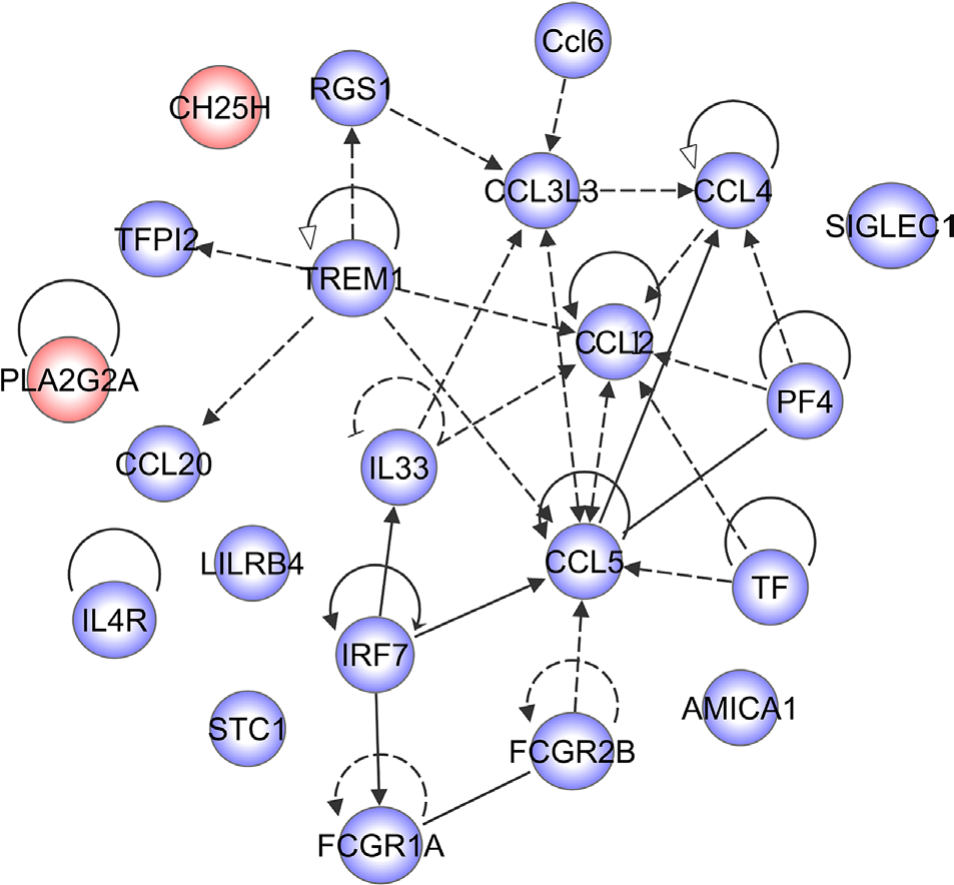
A reconstructed gene network was created using the Ingenuity Pathway Analysis Software (IPA) on the basis of integrated DEGs

To gain insight into the mechanism of molecular changes following blockade of CD74, we performed ingenuity pathway analysis (IPA) for the DEGs integrated at 12, 24 and 48 h. A reconstructed gene network was created, identifying that Ch25h and Pla2g2a were exclusively highlighted as the prominent regulators, without a link with chemokines of the pathways (Figure 8). Both Ch25h and Pla2g2a have been found to act as an amplifier of inflammatory signaling, transcriptionally up-regulated in macrophages or glial cells by pro-inflammatory cytokines [27, 28]. The data provide a previously unrecognized mechanism to explain critical proinflammatory action of MIF in astrocytes, suggesting new approaches for the modulation of innate immune responses.

**Figure 9:**
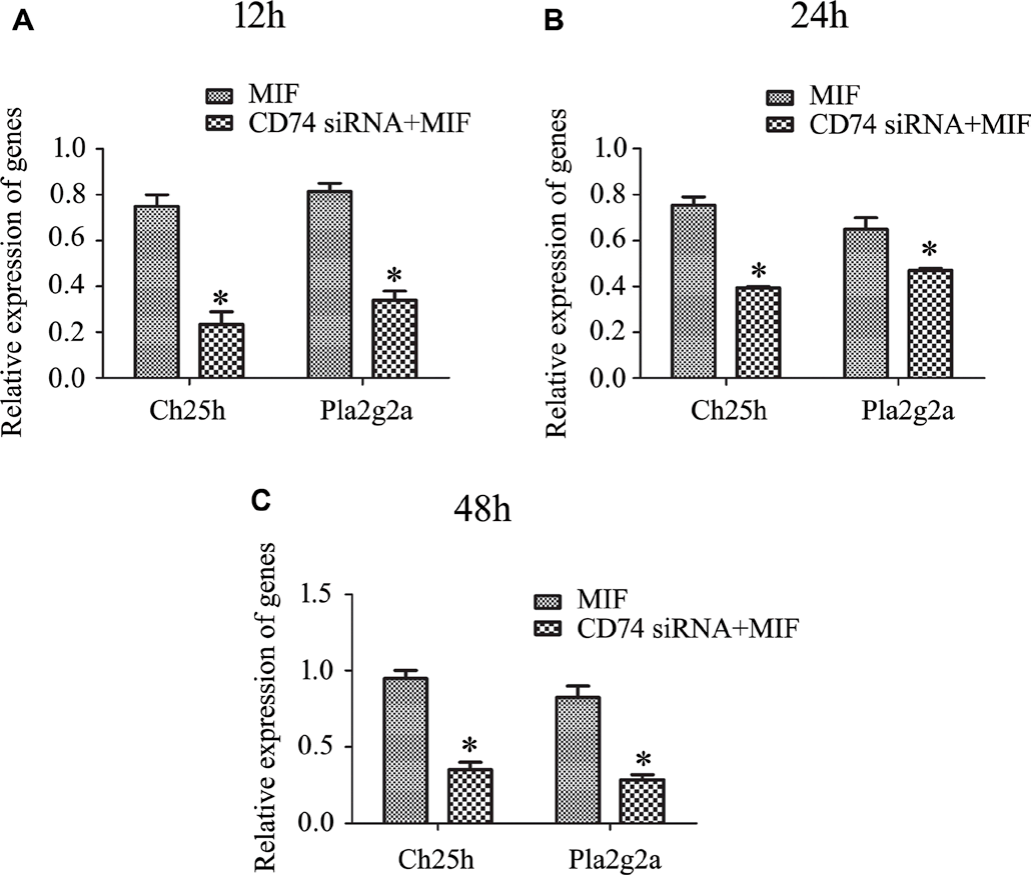
RT-PCR analysis of Ch25h and Pla2g2a expression in primary astrocytes following knockdown of CD74 for 48 h, and then treated with 2 μg/ml recombinant MIF at 12 h (A), 24 h (B) and 48 h (C), respectively.

To verify the inference drawn from transcriptome analysis, RT-PCR was performed to detect the dynamic changes of Ch25h and Pla2g2a, using primary astrocytes treated in the method mentioned above. The results showed a consistency with those of transcriptome analysis (Figure 9), indicating that the molecules downstream of MIF/CD74 axis in the network are potentially implicated in the mediating inflammatory response of astrocytes.

## DISCUSSION

The main functions of astrocytes include regulating extracellular pH and K^+^ levels, removing and recycling potentially toxic glutamate, modulating synaptic activity and blood flow, and influencing the formation and maintenance of the blood-brain barrier (BBB) [1]. In the context of traumatic injury or neurodegenerative diseases, astrocytes undergo reactive astrogliosis that affects functional recovery by formation of permanent glial scars. However, astrocytes are also recognized as immunocompetent cells in the CNS [8, 29]. They binds with endogenous danger signals that are typically released by necrotic cells to alert the immune system, through specific pattern-recognition receptors including TLRs [8]. Activation of these receptors promotes the expression and secretion of several mediators, such as cytokines IL-1, IL-6, IL-10, IFNγ, GM-CSF, TGFβ and TNFα; and the chemokines CCL2, CCL5, CCL20, CXCL10, CXCL12, CXCL1, CXCL2 and CX3CL1 [29]. As such, astrocytes, representing key effectors of the neuroinflammatory response in the pathological conditions.

MIF plays key roles in inflammatory and immune-mediated diseases [10]. It also promotes angiogenensis and tumor progression by regulation of cell proliferation and adhesion [26, 30]. MIF acts by binding to CD74 receptor, thereby activating intracellular signal pathways. The chemokine receptors CXCR2 and CXCR4 have also been shown to interact with MIF, facilitating recruitment of inflammatory cells [17, 19]. MIF-induced ERK1/ERK2 activation was required for the signal transduction and the expression of downstream effector molecules [10]. Also, the protein mediates a rapid and transient activation of the Akt and JNK pathway through CD74 receptor and the intracellular kinases Src and PI3K [23, 31, 32]. In the present study, we have shown that MIF is able to induce phosphorylation of ERK1/ERK2 through CD74 receptor in the astrocytes, suggesting a conserved regulatory mechanism of MIF in different cell types. Whether the interaction of MIF with CXCR2 or CXCR4 receptor was able to activate ERK1/ERK2 pathway in the astrocytes deserves further study.

MIF has been found ubiquitously expressed in the different cell types of CNS and PNS, such as neuron, microglia and glial cells [15, 33, 34]. The cytokine has been released to carry out function through paracrine and autocrine action [23, 35]. The protein can also enter the CNS from blood vessels after destruction of BBB [36]. Thus, in the context of neuropathology, glial cells will meet MIF challenges from different cell types, including themselves. Extensive studies showed that MIF induced cell death and aggravated neurologic deficits, and deletion of MIF or inhibition with antagonist attenuated neuronal death and promoted functional recovery after spinal cord or sciatic nerve injury [16, 37–39]. However, the underlying mechanisms about MIF regulation on neurologic deficits are not completely clarified. Notably, it has been proposed that lack of MIF in mice does not affect hallmarks of the inflammatory responses during the first week after stroke [40]. The effect of MIF assumedly depends on the experimental models, the type of cellular stimulus and spatial-temporal context of MIF expression [40]. After contusion of spinal cord, MIF contributed to the inflammatory response of astrocytes through binding to CD74 receptor, providing a new clue for the production of excessive inflammation.

Transcriptome analysis identified inflammation-related factors, Ch25h and Pla2g2a, downstream of MIF/CD74 axis in astrocytes. Ch25h is an enzyme that hydroxylates cholesterol at the 25 position to generate 25-hydroxycholesterol (25HC), which amplifies the transcriptional response to inflammatory signaling [27, 41]. Ch25h is strongly induced in macrophages by TLRs activation [27, 41]. Agonist of TLR3 (polyI:C) and TLR4 (LPS) significantly stimulated the transcription of Ch25h, whereas least effects for agonist of TLR1/2 (PAM3) [27]. Interestingly, MIF is able to upregulate expression of TLR4 of macrophage response to LPS stimuli [12], indicating an intersection between MIF/CD74 and TLR4 signaling in modulation inflammatory response of astrocytes. Pla2g2a is involved in mediating innate and adaptive immunity, and is upregulated in rat brain after cerebral ischemia and in human Alzheimer’s disease brain [42, 43]. Furthermore, *in vitro* studies with cultured astrocytes demonstrated that Pla2g2a was induced by inflammatory cytokines, such as IL-1β and TNF-α, dependent on ERK1/2 and PI3K pathway. Nevertheless, whether transcriptional synthesis of Pla2g2a is regulated by other signaling cascades has not been explored in sufficient detail. In the present study, we displayed that MIF/CD74 axis was able to mediate expression of Pla2g2a. Whether phosphorylation of ERK1/2 or PI3K is implicated in activation of Pla2g2a deserves further clarification.

In conclusion, cytokine MIF was able to elicit inflammatory response of astrocytes through interaction with CD74 receptor, and activation of ERK was necessary for the intracellular signal transduction. Inflammation-related factors, Ch25h and Pla2g2a, were identified to be potentially implicated in the mediating inflammatory response of astrocytes downstream of MIF/CD74 axis. Our data provide a new target for the control of inflammation following spinal cord injury.

## MATERIALS AND METHODS

### Animals

Adult male Sprague Dawley (SD) rats, weighing 180–220 g, were provided by the Center of Experimental Animals, Nantong University. All animal care, breeding and testing procedures were approved according to the Animal Care and Use Committee of Nantong University and the Jiangsu Province Animal Care Ethics Committee. All animals were housed in individual cages in a temperature and light/dark cycle controlled environment with free access to food and water.

### Establishment of contusion SCI rat model

Rats were anesthetized with an intraperitoneal injection of 10% chloral hydrate (3 mg/kg). The fur was shaved from the surgical site and the skin was disinfected with chlorhexidine. A 15-mm midline skin incision was made to expose the vertebral column. After the spinal thoracic region was exposed by separation of dorsal muscles to the side, the spinous processes of T8–T10 vertebrae were exposed. A laminectomy was performed at vertebral level T9, exposing the dorsal cord surface with the dura remaining intact. The exposed spinal cord segment (about 3 mm in length) was subjected to a moderate vertical impacting load in medial position, using a modified Allen’s weight drop apparatus (8 g weight at a vertical height of 40 mm, 8 g × 40 mm). The impact rod was removed immediately, and the wound was irrigated. Muscles and incisions were sutured using silk threads. Postoperative care included butorphanol administration twice a day for a 5-day period, as well as vitamins, saline, and enrofloxacin twice a day for a 7-day period. Manual expression of bladders was performed twice a day until animals recovered spontaneous voiding.

### Cell culture

Rat spinal cord astrocytes were prepared from newborn Sprague–Dawley rats, 1–2 days after birth, and the astrocytes were isolated and cultured according to previously described methods [44]. Briefly, the cells were enzymatically dissociated using 0.25% trypsin (Gibco-BRL) for 6 min at 37°C, and the suspension was then centrifuged at 1500 rpm for 5 min and cultured in 1:1 Dulbecco’s modified Eagle’s medium: Ham’s F-12 medium supplemented with 10% fetal bovine serum (FBS), 0.224% NaHCO_3_, and 1% penicillin/streptomycin in the presence of 5% CO_2_. A monolayer of astrocytes was obtained 12–14 days after the plating. Non-astrocytes such as microglia were detached from the flasks by shaking and were removed by changing the medium. Third or fourth passage cells were rendered quiescent through incubation in medium containing 0.5% FBS for 4 days prior to the experiments. Confirmation of an astrocyte phenotype was based on the cells exhibiting a characteristic morphology and positive staining for the astrocytic marker glial fibrillary acid protein (GFAP).

### Western blot

Protein was extracted from cells with a buffer containing 1% SDS, 100 mM Tris-HCl, 1 mM PMSF, and 0.1 mM β-mercaptoethanol, following treatment with 0–2.5 μg/ml rat recombinant MIF (ProSpec) for 24 h. Alternatively, protein was extracted from 1 cm spinal segments (*n* = 8) of injured site at 0 d, 1 d, 3 d and 1 week following contusion. Protein concentration of each specimen was detected by the Bradford method to maintain the same loads. Protein extracts were heat denatured at 95°C for 5 min, electrophoretically separated on 10% SDS-PAGE, and transferred to PVDF membranes. The membranes were subjected to the reaction with a 1:1000 dilution of primary antibodies in TBS buffer at 4°C overnight, followed by a reaction with secondary antibody conjugated with goat anti-rabbit or goat anti-mouse HRP dilution 1:1000 (Santa Cruz) at room temperature for 2 h. After the membrane was washed, the HRP activity was detected using an ECL kit. The image was scanned with a GS800 Densitometer Scanner (Bio-Rad), and the data were analyzed using PDQuest 7.2.0 software (Bio-Rad). β-actin (1:5000) was used as an internal control. Antibodies used in Western blot are: MIF (Santa Cruz); p65NFκB, p-ERK1/2, ERK1/2 (CST); CD74 (Biorbyt) and β-actin (Proteintech).

### ELISA

Primary astrocytes were treated with 0–2.5μg/ml rat recombinant MIF for 24 h. Cell supernatants were harvested, and cells were lysed in the buffer containing 1% SDS, 100 mM Tris-HCl, 1 mM PMSF, and 0.1 mM β-mercaptoethanol. The lysates were centrifuged at 12,000g for 15 min. Levels of TNF-α and IL-1β were assessed using the appropriate ELISA kits (BD Biosciences) according to the manufacturer’s directions. Plates were read using a 96-well plate reader (Biotek Synergy2) at a 450 nm wavelength.

### Tissue immunohistochemistry

The vertebra segments were harvested, post-fixed and sectioned. Sections were allowed to incubate with ployclonal MIF antibody (1:100 dilution), polyclonal rabbit anti-IBA-1 antibody (1:200 dilution, Wako), or polyclonal mouse anti-human GFAP antibody (1:200 dilution, Abcam) at 4°C for 36 h. The sections were further reacted with the FITC-labeled secondary antibody goat anti-mouse IgG (1:400 dilution, Gibco), or the TRITC-labeled secondary antibody donkey anti-rabbit IgG (1:400 dilution, Gibco) at 4°C overnight, followed by observation under a confocal laser scanning microscope (Leica, Heidelberg, Germany).

### Immunoprecipitation

The primary astrocytes were washed twice with cold phosphate-buffered saline and then extracted with lysis buffer (20 mM Tris-HCl, pH 7.5, 150 mM NaCl, 1 mM EDTA, 1 mM EGTA, 1% Triton X-100, 2.5 mM sodium pyrophosphate, 1 mM β-glycerolphosphate, 1 mM Na_3_VO_4_, 1 mM phenylmethylsulfonyl fluoride, and Roche Applied Science’s complete protease inhibitors). Whole cell extracts were centrifuged at 14,000 rpm for 20 min to remove the debris. The proteins in the supernatant were measured using a Protein Assay Kit II (Bio-Rad). For immunoprecipitation analysis, 500 μg of total cell lysates was precleared with protein A plus G-Sepharose before incubation with specific antibodies, followed by addition of protein A plus G-Sepharose. The precipitated proteins were resolved in 2 × SDS-PAGE sample buffer and separated by electrophoresis on 10-12% SDS-PAGE. After transferred onto a polyvinylidene difluoride membrane (Millipore Corp.), they were incubated with Anti-MIF or Anti-CD74 antibody and further with horseradish peroxidase-conjugated secondary antibody (Santa Cruz).

### Quantitative real-time polymerase chain reaction (Q-PCR)

Total RNA was prepared with Trizol (Gibco, USA) from cells treated with 0–2.5μg/ml recombinant MIF, and with 40 nM CD74-siRNA (Invitrogen) using a LipofectamineTM RNAiMAX transfection kit (Invitrogen), for 24 h. Fluorescently tagged control Cy3 was used as a marker for evaluation of transfection efficiency. The first-strand cDNA was synthesized using Omniscript Reverse Transcription Kit (QIAGEN) in a 20 μl reaction system containing 2 μg total RNA, 0.2 U/μl M-MLV reverse transcriptase, 0.5 mM dNTP mix, 1 μM Oligo-dT primer. The cDNA was diluted 1:5 before use in Q-PCR assays. The sequence-specific primers were designed and synthesized by Invitrogen (Shanghai, China). Primer pairs for TNF-α: forward primer 5′- CAA ACC ACC AAG CGG AG -3′, reverse primer 5′- GGT ATG AAA TGG CAA ATC G -3′; for IL-1 β, forward primer 5′- CGT CCT CTG TGA CTC GTG G -3′, reverse primer 5′- TCG TTG CTT GTC TCT CCT T -3′; for Ch25h, forward primer 5′- AGC ATA AGG ACG GGA GAG -3′, reverse primer 5′- GCA GAA GCC CAG GTA AGT -3′; for Pla2g2a, forward primer 5′- GCT TCT ACG GTT GCC ATT -3′, reverse primer 5′- GAG TCA CAC AGC ACC AAT CT-3′. Q-PCR reactions were performed in a final volume of 20 μl (1 μl cDNA template and 19 μl Q-PCR reaction buffer containing 2.5 mmol/L MgCl_2_, 0.2 mmol/L dNTPs, anti-sense and sense primers 0.5 μmol/L, taqman probe 0.4 μmol/L, DNA polymerase 0.2 μl and 1 × DNA polymerase buffer). The Rotor-Gene 5 software (Corbett Research, Rotor-Gene, Australia) was used for real-time PCR analysis. Reactions were processed using one initial denaturation cycle at 94°C for 5 min followed by 40 cycles of 94°C for 30 sec, 60°C for 30 sec and 72°C for 30 sec. Fluorescence was recorded during each annealing step. At the end of each PCR run, data were automatically analyzed by the system and amplification plots obtained. MIF full-length plasmid was used to prepare standard curves and used as a specificity control for real-time PCR. The expression levels were normalized to an endogenous β-actin. In addition, a negative control without the first-strand cDNA was also performed.

### Cell proliferation assay

Primary astrocytes were resuspended in fresh pre-warmed (37°C) complete medium, counted and plated at a density of 2 × 10^5^ cells/ml on 0.01% poly-L-lysine-coated 96-well plates. Following treatment with 0–2 μg/ml recombinant MIF for 24 h, 50 mM EdU was applied to the cultures and the cells were grown for an additional 2 hours. Finally, the cells were fixed with 4% formaldehyde in PBS for 30 min. After labeling, the cells were assayed using Cell-Light EdU DNA Cell Proliferation Kit (Ribobio) according to the manufacturer’s protocol. Analysis of astrocytes proliferation (ratio of EdU^+^ to all cells) was performed using images of randomly selected fields obtained on a DMR fluorescence microscope (Leica Microsystems, Bensheim, Germany). Assays were performed three times using triplicate wells.

### Sequencing of mRNA

Total RNA of astrocytes following treatment with CD74-siRNA or scramble for 48 h, and then with 2.0 μg/ml recombinant MIF for 12 h, 24 h and 48 h respectively, was extracted using the mirVana miRNA Isolation Kit (Ambion, Austin, TX) according to the manufacturer’s instructions. They were then selected by RNA Purification Beads (Illumina, San Diego, CA), and undergone library construction and RNA-seq analysis. The library was constructed by using the Illumina TruSeq RNA sample Prep Kit v2 and sequenced by the Illumina HiSeq-2000 for 50 cycles. High-quality reads that passed the Illumina quality filters were kept for the sequence analysis.

### Bioinformatics analysis

Differentially expressed mRNA was designated in criteria of greater than 2-fold or less than 2-fold change in comparison with control. Function of genes was annotated by Blastx against the NCBI database with E-value threshold of 10^−5^. Gene ontology (GO) classification was obtained by WEGO (http://wego.genomics.org.cn/cgi-bin/wego/index.pl) via GO id annotated by Perl and R program. Kyoto Encyclopedia of Genes and Genomes (KEGG) pathways were assigned to the sequences using KEGG Automatic Annotation Server (KAAS) online. For all heatmaps, genes were clustered by Jensen-Shannon divergence.

To understand the mechanism of molecular changes, a reconstructed gene network was created using the Ingenuity Pathway Analysis Software (IPA) on the basis of differentially expressed genes (fold change < 0.5 for downregulated genes at 12 h, 24 h and 48 h following CD74 knockdown, to investigate their regulatory pathways and cellular functions [45].

### Statistical analysis

Statistical significance of differences between groups was analyzed by one-way analysis of variance (ANOVA) followed by Bonferroni’s post-hoc comparisons tests with SPSS 15.0 (SPSS, Chicago, IL, USA). Normality and homoscedasticity of the data were verified before any statistical analysis using levene’s test. Statistical significance was set at *p* < 0.05.
